# An Overview of Major *Penicillium* Species Associated with Plant Diseases

**DOI:** 10.3390/jof12040286

**Published:** 2026-04-17

**Authors:** Latiffah Zakaria

**Affiliations:** School of Biological Sciences, Universiti Sains Malaysia, Gelugor 11800 USM, Penang, Malaysia; Lfah@usm.my or latiffahz@yahoo.com

**Keywords:** *Penicillium*, plant disease, postharvest rot, blue mold, green mold, agricultural crops

## Abstract

Species of *Penicillium* are among the most important fungal pathogens responsible for postharvest diseases of agricultural crops worldwide. This review provides an overview of five economically important *Penicillium* spp., namely *P. expansum*, *P. digitatum*, *P. italicum*, *P. citrinum*, and *P. oxalicum*. Emphasis is placed on *P. expansum*, *P. digitatum*, and *P. italicum* which are the main causal agents of blue mold and green mold rots in pome fruits and citrus, commodities that dominate global fresh produce trade and long-term storage. While studies on plant-pathogenic *Penicillium* are mainly focused on these hosts, this review highlights reports of infections in other crops across diverse geographic regions, highlighting the broader host range of these species. The main aspects highlighted include host specificity and diversity, production of mycotoxins and other secondary metabolites, current management and control strategies, and the potential influence of climate change on disease incidence and severity. Understanding the biology and epidemiology of plant-pathogenic *Penicillium* species is essential, as several species are both pathogens and producers of mycotoxins, leading to quality deterioration and nutrient depletion resulting in economic losses.

## 1. Introduction

*Penicillium* is one of the most common fungi occupying various substrates as a saprophyte and pathogen in the soil, plant residues, water, outdoor and indoor environments, on a variety of food products, and as contaminants on food crops [1]. As such many *Penicillium* species have positive and negative impacts on humans. Negative impacts include causing food spoilage, as a pathogen of pre- and postharvest diseases and as a mycotoxin producer that can affect human and animal health [2]. There are also many positive impacts of *Penicillium* such as production of various types of biocompounds for medical purposes, production of enzymes for industrial applications and use in the food industry [3,4,5].

Various *Penicillium* species have been reported in several tropical ecosystems [6,7,8,9,10], indicating that *Penicillium* can thrive in different tropical environments, which may be due to their adaptability and flexibility to high humidity, high temperature, and availability of suitable substrates. Therefore, postharvest agricultural products in the tropics are susceptible to *Penicillium* infection.

*Penicillium* species are widely known as postharvest pathogens of fruit crops, particularly pome fruits and citrus. In addition to fruit crops, *Penicillium* also infects vegetables, bulbs, cultivated mushrooms, herbs, corn, onions, and garlic, causing blue mold and/or green mold rot, which can lead to economic losses. Some pathogenic *Penicillium* spp. produce mycotoxins that contaminate fruits and products derived from the fruit crops.

Plant-pathogenic *Penicillium* are wound pathogens that have a necrotrophic phase, during which the pathogen infects fruits through wounds that can be in the form of bruises, punctures or cracks, caused by mechanical means during harvesting and postharvest handling [11]. Insect infestation and damage by heavy rain or storms are also factors contributing to injury to the fruits. However, injuries during fruit picking and handling processes are the main entry points of the pathogen conidia [12]. *Penicillium* produces cell wall-degrading enzymes and toxic secondary metabolites that break down host tissues and weaken structural defenses. Once the host tissues die, the pathogen switches to the saprophytic phase, deriving nutrients from the dead tissues, which facilitates growth and colonization of the host plant [13,14]. Due to the dual lifestyle, *Penicillium* can grow and thrive in both natural and postharvest environments, enhancing its success as a postharvest pathogen.

Conidia of *Penicillium* are airborne and often found in storage facilities, in collection or orchard bins, and on floors. When the collection bins containing the fruits are immersed in a water tank, the conidia are released into the water flume, carried through the water, and infect wounded fruits [15]. During cold storage, lesions on infected fruits develop slowly, and the fungi produce abundant conidia. The conidia are disseminated through the air and infect other susceptible fruits, and some of the conidia may stick to the walls and floor of the storage facility [15].

This review focuses on postharvest rot caused by *Penicillium* species within storage and packinghouse contexts, with particular attention to food safety concerns. Among these, *P. expansum*, *P. digitatum*, and *P. italicum* are recognized as the major pathogens responsible for blue mold and green mold rot of pome fruits and citrus. Additionally, *P. citrinum* and *P. oxalicum* are highlighted as opportunistic postharvest pathogens that are relevant to food safety considerations and contribute to the economic impact of postharvest losses.

Studies on plant-pathogenic *Penicillium* predominantly focus on pome fruits and citrus as these commodities are traded worldwide and kept in storage for long periods of time. Therefore, in this overview, other crops or plants infected by the five *Penicillium* spp. which have been reported in several countries worldwide are also highlighted. The emphasis of this overview is on the host range, production of mycotoxins and other secondary metabolites, management approaches and effects of climate change. The information on *Penicillium* spp. associated with agricultural crops is important as a few species are destructive plant pathogens and mycotoxin producers. Infection by *Penicillium* on agricultural crops can cause defects and nutrient depletion, causing economic losses.

## 2. Major Plant-Pathogenic *Penicillium*

*Penicillium* species associated with plant diseases are primarily known to occur postharvest and are well-adapted to storage conditions. Typical signs and symptoms of the rot disease associated with *Penicillium* include soft rot, discoloration, and conidial production in the infected tissues, although they might vary according to the host, environmental conditions, and the specific species involved [16].

### 2.1. Penicillium expansum

*Penicillium expansum* is the main pathogen causing blue mold rot on apples and pears. Early symptoms appear as soft, watery lesions that are light brown. As the lesions mature, the conidia turn blue-green. Due to the soft, watery appearance of rotted tissue, the disease is often called soft rot. The lesions have a clear boundary between the diseased and healthy areas. Rotted tissues can easily be separated from healthy tissues, often leaving a bowl-like cavity. The surface of older lesions may be covered with blue-green conidial tufts. Rotten fruit also emits an earthy and musty smell [15,16].

Temperature influences the growth of *P. expansum,* which can grow at temperatures ranging from −2 °C or even lower up to 35 °C, and can survive with a water activity as low as 0.83 [17]. The optimal temperature for growth is 25 °C [17,18], and it thrives best at temperatures between 15 and 25 °C, with water activity ranging from 0.960 to 0.980 [18]. According to Tannous et al. [19], the optimal growth *P. expansum* was 0.92 cm per day at 24 °C, with a pH of 5.1 and a high-water activity of 0.99. The growth of *P. expansum* was reduced at pH 2.5 and pH 8 [20]. Within the optimal temperature range, the effect of slight temperature variation on the fungal growth is less significant than that at lower temperatures, suggesting that even a minor change in storage temperature can greatly affect the shelf life of pome fruit [18].

The ability of *P. expansum* to thrive effectively within the temperature range of 15–25 °C, as well as at 5 °C, accounts for its considerable effect during the prolonged storage of pome fruits and other types of fruits [18]. *Penicillium expansum* has the ability to adapt to refrigerated or cold storage that slows down metabolic activities without completely stopping the growth. However, ensuring that fruits maintain their turgor often requires high humidity, which promotes fungal growth. This indicates the need for a careful balance between managing storage conditions and the implementation of suitable disease management strategies.

### 2.2. Host Range of Penicillium expansum

*Penicillium expansum* is recognized as one of the most economically important postharvest pathogens due to its aggressive colonization of fruit tissues, ability to grow at low temperatures, and production of patulin. Although apples and pears are the main hosts, *P. expansum* has a wide host range, infecting numerous fruit crops [21,22].

On apple and pear, soft rot caused by *P. expansum* spreads rapidly due to degradation of the fruit tissues and conidia production, with the appearance of blue-green conidial masses. Apple and pear account for the majority of infections reported worldwide and are of particular concern due to patulin contamination, particularly in the products derived from both fruits, which can lead to food safety risks [23,24,25]. Quince is also susceptible to *P. expansum* infection [14,26].

*Penicillium expansum* has been reported to cause rot in peach, nectarine, apricot, cherry, strawberry, grape, plum, citrus, kiwifruit, persimmon, and pomegranate (Table 1). In these fruit crops, infection typically occurs through wounds inflicted during harvest and handling, and disease development is favored in high-humidity conditions [14,27]. Reports of *P. expansum* infection have also been reported on the rhizome of medicinal herbs, *Polygonatum odoratum* var. *pluriflorum*, onions, ornamental bulbs of iris and tulip, and vine legumes (*Apios mericana*) (Table 1).

*Penicillium expansum* infection on various crops represents a notable agricultural risk. The ability to infect various crops or plants suggests a higher potential for inoculum accumulation in different agricultural settings, along with greater risks of cross-contamination in processing and storage facilities. This highlights the need for a more thorough and integrated strategy for blue mold rot management in various agricultural commodities.

### 2.3. Factors Contributing to the Wide Host Range of P. expansum

*Penicillium expansum* is recognized for its ability to infect a wide range of host plants, a trait that is supported by its diverse genomic and physiological features. This postharvest rot pathogen uses several virulence factors that combines tissue breakdown, changes in metabolism, and suppression of host defense. The main virulence factors include the production of cell wall-degrading enzymes, secondary metabolites, small secreted proteins, and stress response systems [14,21,63].

The genome of *P. expansum* contains a higher number of genes encoding carbohydrate-active enzymes (CAZymes) and is abundant in cell wall-degrading enzymes responsible for the synthesis, degradation, and modification of carbohydrates in host plants. Genome analysis of CAZymes revealed that *P. expansum* encodes fewer cellulose-degrading enzymes than those that degrade hemicellulose and pectin. Within the CAZyme families, polysaccharide lyase, glycoside hydrolase, and carbohydrate esterase families are involved in pectin degradation [64,65]. *Penicillium expansum* also possesses a higher number of carbohydrate esterases, glycoside hydrolases, and polysaccharide lyases, which may be attributed to its larger genome size compared to *P. italicum* and *P. digitatum*. These enzymes are associated with the ability to utilize diverse carbohydrates present in the host plant environment [14,64]. Consequently, the greater number of CAZyme-related genes in *P. expansum* might partly explain its ability to infect a broader range of host plants.

*Penicillium expansum* is capable of producing a range of secondary metabolites, including patulin and citrinin, which can damage host tissues and suppress host defenses, hence promoting colonization. Its genome contains several gene clusters associated with secondary metabolism that are linked to its pathogenic properties [19,21]. *Penicillium expansum* has the highest number of secondary metabolite gene clusters compared to *P. italicum* and *P. digitatum*. In the genome of the *P. expansum* T01 strain, 71 genes associated with secondary metabolite backbones and 55 secondary metabolite clusters have been identified, suggesting that *P. expansum* possesses the capability to produce a diverse range of secondary metabolites [65,66,67].

The production of patulin is associated with the virulence of *P. expansum* as shown by the upregulation of the patulin biosynthetic gene cluster during colonization of host plants, which contributes to host susceptibility and tissue maceration. Although patulin is not essential for infection, the mycotoxin facilitates disease development by inducing oxidative stress in the host, leading to loss of cell membrane integrity and disrupting defense signaling pathways. Patulin functions synergistically with cell wall-degrading enzymes and other secreted factors to intensify aggressive rotting of the host plant [19,67,68,69]. *Penicillium expansum* possesses a greater number of secondary metabolite gene clusters and pathogenic genes, including a complete patulin cluster, than *P. italicum* and *P. digitatum*. These findings may provide insight into the molecular mechanisms underlying patulin biosynthesis and host specificity in *P. expansum* [21,64,66].

*Penicillium expansum* expresses a wide range of secreted effector proteins, lectins, proteases, and glucanases, which facilitate defense suppression and tissue maceration [63]. The secreted proteins produced by plant pathogens can influence the development and success of an infection [1,69,70,71]. Notably, *P. expansum* possesses a greater number of genes encoding secreted proteases than *P. italicum* and *P. digitatum*. Diverse secreted proteases are advantageous as they enable the fungus to exploit a variety of nutrients and counteract the protein-based defense mechanisms of the host [64].

*Penicillium expansum* secretes substantial quantities of organic acids, including gluconic acid, glucose oxidase, citric acid, and fumaric acid, which function as virulence factors [67,72]. These organic acids contribute to acidification of the host tissue environment, thereby altering the biochemical processes of the host. The acidification of the host tissues enhances the activity and expression of enzymes that degrade cell walls, compromises the host defense mechanisms, facilitates nutrient mobilization, and results in tissue necrosis. These combined effects promote the penetration, spread, and nutrient uptake of fungi [14,73,74].

The ability of *P. expansum* to grow under various environmental conditions, particularly at low temperatures during storage, combined with its highly adaptable regulatory networks involved in virulence and stress responses, facilitates the pathogen’s capacity to infect a broad range of host plants [21,29,65].

### 2.4. Mycotoxin Production of P. expansum

*Penicillium expansum* is the primary producer of patulin [75]. Patulin production is highly dependent on both the specific strain of the pathogen and environmental factors such as substrate, temperature, and storage conditions [76]. Most *P. expansum* isolates examined in various studies have been shown to produce patulin both in vitro and in decayed fruit. Patulin is heat-resistant and can remain stable during processing [76,77].

Infection of fruit crops by *P. expansum* during storage is the primary cause of patulin contamination. The pathogen’s ability to thrive at 0 °C makes it a major contributor to fruit crop loss during storage [77]. As the duration of storage increases, the degradation of fruit tissues occurs, resulting in the accumulation of considerable amounts of patulin. The presence of patulin in rotten fruits can also affect the products derived from them, such as juice, jam, nectar and dried fruits, particularly when these products are produced from a combination of rotted and healthy fruits [78]. If rotten fruits are not removed during processing, patulin may contaminate the fruit products, resulting in toxicological risks to their consumption [79].

The presence of patulin in various fruit products, including juices, dried fruits, jams, baby foods, and purees, has been reported worldwide. Patulin in apple juice has been reported in Italy [80], Malaysia [81], Tunisia [82,83] and Iran [84]. Other than apple juice, patulin has been detected in grape and orange juices [85,86]; pear, apricot, peach, and mixed juices [80]; and pomegranate juices [84]. Patulin has also been reported in mangoes, oranges, and fruit-derived products, including juice, jam, and pulp [86]. In addition, patulin contamination occurs in dried figs, seedless dried longans, dried hawthorn [87], various cultivars of citrus fruits [88], olives, and olive-based products [89]. There is also the possibility of patulin contamination in stored onions, Dunggulle rhizomes (*Polygonatum odoratum*), and *Apios americana* tuber, as *P*. *expansum* has been reported to cause postharvest rot in these crops [59,60,62].

The presence of patulin in various fruit-derived products is due the stability of the mycotoxin under acidic conditions, as it is not eliminated during thermal processing [90]. Patulin production in fruits may be attributed to pH, temperature, fruit cultivar, gas composition, and water activity, in which pH plays an important role [20,91]. Various ambient pH values not only influence patulin production, but also affect the sporulation, growth, and biomass of *P. expansum*, as well as the genes involved [20,92,93].

Citrinin is also produced by *P. expansum*, but the production is influenced by both the strain and substrate, similar to patulin production. An in vitro study conducted by Viñas et al. [94] revealed that a number of *P. expansum* strains found in apple packaging can produce citrinin, with the majority (73.2%) of citrinin-producing strains originally isolated from rotting apples. Many *P. expansum* strains isolated from natural environments have been reported to produce patulin and citrinin [94,95]. Citrinin is frequently found together with patulin in infected fruits and apple juice [96]. A study conducted by Martin et al. [97] found that patulin and citrinin are present in 19.6% of rotted apples, with patulin occurring at much higher concentrations than citrinin. Citrinin levels were usually low, ranging from 0.32 to 0.92 mg/kg, while patulin levels varied considerably among different apple varieties. *Penicillium expansum* has also been reported in grapes and is capable of producing both patulin and citrinin. Both mycotoxins have been detected in naturally contaminated grape must, although wine made from these grapes showed patulin concentrations below the detection limits [98]. The presence or co-occurrence of both patulin and citrinin in naturally infected fruits, as well as their combined toxicity, can have an additive effect [99].

During host colonization by *P. expansum* in apples, citrinin was detected at a later stage. Although citrinin has been suggested to contribute to apple colonization, it does not appear to be a critical factor but rather an accessory factor and is influenced by environmental conditions, specific fungal strain, and apple varieties [100]. In a study by Heider et al. [101], negligible levels of citrinin were detected in apples stored for an extended period of 11–12 days. Citrinin production may increase when *P. expansum* encounters oxidative stress conditions, suggesting that the metabolite may play a protective role due to its antioxidative properties.

In addition to patulin and citrinin, various secondary metabolites have been identified in *P. expansum* isolates, including chaetoglobosins, communesins, roquefortine C, and expansolides A and B [99,102]. *Penicillium expansum* constantly produces expansolides A and B in rotten apple fruits. This suggests that spoiled apples may contain expansolides A and B, together with patulin and citrinin [102]. In addition to the secondary metabolites reported by Andersen et al. [99] and Watanabe [102], Shen et al. [103] also listed additional secondary metabolites produced by *P. expansum*, which include, among others, aurantioclavine, rugulovasine, penostatins I, N-acetyltryptamine, geosmin, cytochalasin A/B, and sydonic acid, as well as expansols A and B.

## 3. *Penicillium digitatum*

Green mold rot affecting citrus fruits caused by *P. digitatum* is one of the most serious postharvest diseases, resulting in losses of at least 10%, and in severe cases, up to 90% [104]. *Penicillium digitatum* is commonly known as a green mold due to the appearance of olive-green conidia, which become apparent as the disease advances. Infections of adjacent citrus fruits are rare; however, conidia have the potential to contaminate fruit surfaces. Damaged tissue with broken oil glands releases several volatile compounds, such as limonene, myrcene, alpha-pinene, and beta-pinene, along with organic acids and sugars that encourage the germination of conidia [105].

*Penicillium digitatum* is capable of growing within a temperature range of 6–7 °C, with a maximum of 37 °C. The optimum temperature for growth is 25 °C [106]. The minimum water activity required for growth at 25 °C is 0.90, increasing to 0.95 at 30 °C and 0.99 at 5 °C [2]. Notably, no germination occurs at 0.87 water activity and at 37 °C [107].

*Penicillium digitatum* can only penetrate citrus fruit tissues when the tissues have been bruised or damaged [108]. Once the pathogen successfully enters the host tissues, it requires optimal temperature and humidity for growth and development. The pathogen releases considerable amounts of cellulase to break down the cell wall after the tissue softens due to pectinase activities, thereby acquiring abundant supply of nutrients for rapid growth [109]. This process also results in rapid spread of soft rot lesions around the wounds on the fruit surface. Cheng et al. [110] suggested that hemicellulase might also be involved in citrus infection by *P. digitatum*.

*Penicillium digitatum* predominantly infects citrus fruits, exhibits strong host specificity, and is highly adapted to grow on these fruit crops [66,111]. However, there are reports indicating that *P. digitatum* also infects nectarine, plum, and ginger (Table 2). Based on a pathogenicity study performed by Louw and Korsten [44], *P. digitatum* is highly pathogenic and aggressive on nectarines and plums, and forms larger rot lesions than *P. expansum* within a shorter time. Rapid rotting of nectarines and plums by *P. digitatum* can potentially cause losses in the stone fruit industry. Ginger soft rot caused by *P. digitatum* was reported by Shakeel et al. [112]; however, identification of the pathogen was based on morphological characteristics.

### Secondary Metabolites of Penicillim digitatum

Infections caused by *P. digitatum* in citrus do not require the production of patulin or other mycotoxins. However, during infection of citrus fruits, *P. digitatum* produces thermogenic alkaloids such as tryptoquialanines A and B, tryptoquivalines, and fumiquinazolines, which may contribute to its pathogenicity [104,115,116]. In a study by Rovetto et al. [117], *P. digitatum* emerged as the most commonly isolated species from mummified and hail-damaged blood oranges (*Citrus* × *sinensis*), and patulin was detected in both the juice and peel of the citrus fruits. However, it was not definitively shown that *P. digitatum* was responsible for producing the detected patulin.

*Penicillium digitatum* has been found to produce various secondary metabolites such as phenylalanine–proline diketopiperazine [116,118], fumitremorgin C, and yanuthone D [103]. It remains uncertain whether these secondary metabolites have toxicological implications for food safety. The availability of the *P. digitatum* genome sequence may facilitate investigation and provide insight into the secondary metabolites produced by this green mold through genome mining of the biosynthetic gene clusters associated with these metabolites [66,119].

## 4. *Penicillium italicum*

*Penicillium italicum* is responsible for blue mold rot on citrus fruits and has also been identified as pathogen of garlic rot [120]. The pathogen is resilient to cold temperatures and low-moisture conditions [105,121]. Additionally, the pathogen can grow and cause fruit rot at temperatures as low as 0 °C, and contributes to serious infection of citrus fruits kept in cold storage, resulting in considerable spoilage during longer storage periods [107]. Moreover, conidia can spread readily, leading to infection of healthy fruits.

The optimal growth for *P. italicum* occurs at 25 °C and water activity levels ranging from 0.96 to 0.98 [122,123]. Both temperature and water activity play a crucial role in determining lesion size, with a notable increase in blue mold rot severity occurring at temperatures between 20 °C and 25 °C when conidia concentration is elevated [123,124]. According to Palou [125], *P. italicum* can germinate and grow at a water activity of 0.87, and it was observed that oranges stored at 4 °C developed symptoms of blue mold rot and green mold rot at 16 and 23 days, respectively. Prusky et al. [126] highlighted that both *P. italicum* and *P. digitatum* can increase their virulence by reducing the pH of the environment surrounding wounds on citrus rind.

Several fruit crops have been listed in the USDA–ARS Fungal Database [127] to be associated with *P. italicum*, including persimmon, banana, melon, mango, avocado, wild cherry (*Prunus avium*), Japanese plum (*Prunus salicina*), grape, and Asian pear (*Pyrus serotina* var. *culta*). Other host plants listed in the database are Ussurian pear (*Pyrus ussuriensis*), an ornamental tree, tomato, sweet potato, tea tree oil (*Camellia oleifera*), *Carica xheilbornii var. pentagona* and wheat (*Triticum vulgare*). However, there is a lack of documented reports of economically important plant diseases attributed to *P*. *italicum* in the listed host plants, suggesting that citrus remains the primary host for this blue mold pathogen [1].

### Secondary Metabolites of P. italicum

Although *P. italicum* is an important blue mold pathogen, it does not produce patulin or citrinin. *Penicillim italicum* was not listed as a mycotoxin producer by Frisvad and Samson [1] but the pathogen produced other metabolites. Various secondary metabolites produced by *P. italicum* may contribute to the interaction between the host and the pathogen, possibly playing a role as antimicrobial compounds that enable the pathogen to thrive in infected crops. These secondary metabolites include, among others, dihydro-4-methoxy-2H-pyran-2-one, verrucolone, formylxanthocillin X, dehydrofulvic acid, PI-3 and PI-4, 4-methoxy-6-n-propenyl-2-pyrone, and 5-hydroxymethyl-2-furic acid [1] as well as revianamide F, dehydrodeoxybrevianamide E, deoxyisoaustamide, and 12,13-dehydroprolyltryptophanyldiketopiperazine [128]. Additionally, other secondary metabolites such as sulochrin, chrysogine, and dichlorodiaportin derivatives showed moderate phytotoxic effects, aiding the pathogen in softening citrus peel tissues during the initial stages of infection [128].

Volatile compounds generated by *P. italicum*, including ethyl acetate, isopentanol, linalool, isobutanol, 1-octene, ethyl butanoate, ethyl 2-methyl-butanoate, 1-nonene, styrene, and citronellene [129], may aid in the dissemination of the pathogen during storage. These volatile compounds are primarily associated with competitive saprophytic growth of *P. italicum* [128].

Similar to *P. digitatum*, the genome of *P. italicum* has also been sequenced; however, biosynthetic gene clusters, particularly those related to polyketide synthases, have not been sufficiently studied [130].

## 5. Factors Contributing to Host Specificity of *P. digitatum* and *P. italicum*

The ability of a fungal pathogen to infect a specific host plant is governed by a set of virulence genes. These genes are crucial in establishing host specificity of plant-pathogenic fungi, as they influence the pathogen’s ability to infect a particular crop or plant species. These genes encode effector proteins and secondary metabolites that modify the host immune response and facilitate colonization. Fungal pathogens produce effector proteins that suppress initial immune response and assist in the infection process. Furthermore, these pathogens generate mycotoxins and carbohydrate-active enzymes (CAZymes) that break down plant tissues. The interaction between virulence genes and host resistance genes affects both the virulence of the pathogen and the host’s defense mechanisms [65,130,131,132]. Genomic analyses of *P. digitatum* and *P. italicum* have identified several virulence factors including effector proteins, secondary metabolites and CAZymes, which may contribute to the host specificity of both pathogens [66,117,132].

### 5.1. Penicilllium digitatum

Genome analysis of *P. digitatum* isolates by Julca et al. [133] revealed low genomic variation and few single-nucleotide polymorphisms, suggesting a recent evolutionary bottleneck or rapid clonal expansion linked to global citrus cultivation. This limited genetic variation is in contrast with the higher genomic variation observed in *P. expansum*. Ballester et al. [21] associated differences in genome variability with a smaller gene content and greater host specificity. Aligned with this, Marcet-Houben et al. [66] reported reduced gene content in *P. digitatum*, reflecting its host specificity.

During infection of citrus fruits, CAZymes increase, and enzymes that break down cell walls are important for the virulence of *P. digitatum* [104,119]. According to a genomic analysis of *P. digitatum*, CAZymes are linked to glycoside hydrolases, carbohydrate esterases, or polysaccharide lyases, which help break down fungal cell walls [66]. Wang et al. [119] found CAZyme genes such as glycoside hydrolases, glycosyl transferases, auxiliary activities, carbohydrate esterases, polysaccharide lyases, and carbohydrate-binding modules, indicating the role of the enzymes in pathogenicity and virulence. The largest groups of genes that increase during citrus fruit infection are those encoding plant cell wall-degrading enzymes and fungal proteases [134]. Costa et al. [104] also noted that the genes controlling cell wall-degrading enzymes are linked to pathogenicity.

Secondary metabolites, including various mycotoxins, are key contributors to the pathogenicity of many plant-pathogenic fungi and potential virulence factors. In *P. digitatum*, multiple secondary metabolite biosynthetic gene clusters have been identified, including the nonribosomal peptide synthases (NRPS), polyketide synthases (PKS), terpene, and hybrid pathways [66,119]. These clusters contain sequences linked to metabolite families, such as chaetoglobosins, naphthopyrone, squalestatin S1, PR-toxin, yanuthone D, tryptoquialanines, and nidulanin A, indicating the ability of the fungus to produce these compounds. Ariza et al. [116] reported major metabolites in *P. digitatum* biomass, including indole alkaloids tryptoquinalanine A and B, and steroids such as cholesterol and ergosterol derivatives, which may influence fungal interactions with citrus tissues. Seven genes of the patulin biosynthetic cluster (patB, patC, patD, patF, patG, patJ, and patL) have been detected; however, this cluster is nonfunctional in *P. digitatum* due to the presence of a truncated patI pseudogene, loss of essential components, and absence of nearby backbone genes [66].

Volatile organic compounds released from infected citrus peels are unique to each species and facilitate the germination of *P. digitatum* conidia as well as the development of germ tubes. These chemical cues play a role in recognizing the host, therefore improving the success of citrus infections [105]. Moreover, mechanically damaged fruits contain higher quantities of limonene and other citrus monoterpenes [116]; these volatile organic compounds may have functions in host recognition.

### 5.2. Penicillium italicum

Based on comparative genomic and omics studies, *P. italicum* occupies an evolutionary position between *P. digitatum* and *P. expansum*. Genomic and transcriptomic analyses revealed that *P. italicum* shares several host-adaptation characteristics with *P. digitatum*, such as reduced gene family complexity in certain CAZymes and virulence-related pathways, while maintaining a broader metabolic and secondary metabolite repertoire [135]. In contrast, *P. italicum* lacks extensive secondary metabolite clusters, patulin biosynthetic genes, and large-scale virulence gene expansions, which are characteristic of *P. expansum* [14,64]. These findings suggest that *P. italicum* represents an intermediate stage in the evolution of *Penicillium’s* host range: it is neither as host-restricted as *P. digitatum* nor as host-diverse as *P. expansum*.

Based on transcriptomic and metabolomic analyses, *P. italicum* possesses a greater number of secondary metabolite biosynthetic gene clusters, averaging 43, than *P. digitatum*, which averages 32 [135]. Certain secondary metabolites, such as hesperetin 7-O-glucoside, naringenin, 3′,5,7-trihydroxyflavanone, diosmin, and brevianamide F, are recognized for their roles in the defense mechanisms of citrus [128]. Nevertheless, none of the secondary metabolites were identified as virulence factors. It is well established that these secondary metabolites contribute to disease development and can suppress or interfere with the defense systems of citrus fruits in various pathogen–host interactions [130].

The main factors contributing to the virulence and colonization of *P. italicum* are facilitated by polygalacturonases, which lead to tissue degradation [126,130]. These enzymes’ activity is more effective under acidic conditions, and since *P. italicum* can lower the pH by accumulating organic acids, particularly citric acid, enzyme activity is enhanced during infection. This suggests that pH serves as a regulator of gene expression, ensuring the expression of genes that code for extracellular enzymes, such as PEPG1 for the polygalacturonase enzyme [126]. Genome analyses have shown that *P. italicum* possesses genes encoding nine CAZyme families that are associated with pectin. Considering that the cell walls of fruit cells contain a substantial amount of pectin, the majority of virulence mechanisms are linked to the modification or degradation of polysaccharides [64].

Secretome analyses of *P. italicum* reveal a wide range of secreted proteins, including CAZymes and other cell wall-degrading enzymes, peptidases, proteins analogous to RNases, and numerous small cysteine-rich candidate effectors. These findings support the view that *P. italicum* employs a variety of mechanisms to establish its virulence. The virulence strategies utilized by *P. italicum* encompass the enzymatic degradation of citrus structural defenses, proteolytic and nucleolytic inhibition of citrus defense mechanisms, and potential modulation of host signaling pathways by effector proteins [64,135,136,137].

As noted by Li et al. [138] and Yin et al. [139], Dicer-type genes, which encode for RNase III-like enzymes, play a vital role in RNA interference and are important for the growth and pathogenesis of *P. italicum*. They highlighted that cross-kingdom RNA interference might serve as a potential virulence mechanism, where small interfering RNAs (siRNAs) are transferred between the fungus and the host plant. In this interaction, a small proportion of *P. italicum* siRNAs can penetrate plant cells and suppress genes associated with defense mechanisms, thereby promoting infection. The majority of siRNAs function within the fungus itself [139,140].

## 6. *Penicillium oxalicum*

*Penicillium oxalicum* is often found in soil and is typically associated with the breakdown of organic materials and plant debris. It has been detected in indoor air, food, and animal feed, and is one of common species isolated from moldy corn [2,141]. This species is xerotolerant and is able to grow under substantial water deficits. *Penicillium oxalicum* is considered mesophilic and has adapted to a wide range of environmental conditions, enabling it to grow in various ecosystems such as soil and air [141].

Depending on the strain, *P. oxalicum* can grow under a broad range of conditions, including temperatures between 25 and 35 °C and a pH tolerance of 5–9 [142]. The strain *P. oxalicum* HYC2101, which is heat-tolerant and entomopathogenic, has a growth temperature range of 10–35 °C [143]. The strain *P. oxalicum* EEEL01, known for its resistance to alkaline conditions, demonstrated a high tolerance to alkalinity (pH 12) and salinity (NaCl 2.0 M), producing substantial amounts of oxalic acid to lower the pH of the medium to pH 2 [144].

Although *P. oxalicum* is not classified as one of the main postharvest pathogens, there have been increasing reports of its occurrence in various fruits and vegetables, particularly under storage conditions. *Penicillium oxalicum* is the primary species responsible for corn ear rot and seedling blight. These conditions mainly occur in corn ears that have been physically wounded or have been affected by insects [145,146,147]. *Penicillium oxalicum* also causes stem rot in cucumbers, stem and fruit rot in tomatoes, fruit rot of musk melon and blue honeysuckle, rot of oranges, tuber rot in yam, leaf spot of pineapples and kiwi, and blue mold rot in *Gastrodia elata* and *Astragalus membranaceus* (Table 3).

*Penicillium oxalicum* can act as both pathogen and biological control agent against other pathogens. The fungus has been identified as a promising biocontrol agent for tomato diseases caused by *Fusarium oxysporum* f. sp. *lycopersici, Verticillium dahliae* [169,170] powdery mildew on strawberry leaves caused by *Sphaerotheca macularis* [171], and wilt and root rot pathogens of pea, including *Fusarium oxysporum, Rhizoctonia solani*, and *Pythium ultimum* [172]. *Penicillium oxalicum* may serve as a biocontrol agent against potato cyst nematodes by decreasing the hatching of juveniles and the number of cysts of *Globodera pallida* [173]. These findings indicate that *P. oxalicum* exhibits a broad range of activity against various plant pathogens.

### Mycotoxins and Secondary Metabolites of P. oxalicum

*Penicillium oxalicum* is not a major producer of mycotoxins in postharvest diseases; however, it produces a range of secondary metabolites. Patulin and citrinin are not produced by *P. oxalicum* and can be used to assist in distinguishing *P. oxalicum* from other *Penicillium* species. Nonetheless, *P. oxalicum* exhibits diverse metabolic capability, producing diverse secondary metabolites including polyketides, indoles, oxaline, and chromones. Many of these secondary metabolites possess bioactive or cytotoxic characteristics [174].

Numerous secondary metabolites are produced by *P. oxalicum* including polyketides, such as oxalichroman A and oxalihexane A [174], oxaline and chromones (isorhodoptilometrin and 5-hydroxy-7-[2′-hydroxypropyl]-2-methyl-chromone) [175], and altersolanol A [176], which exhibit cytotoxic properties. These metabolites may play a role in the adaption of *P. oxalicum* within its environment as well as in deterring or inhibiting the growth of other microorganisms.

*Penicillium oxalicum* produces a toxic secondary metabolite, secalonic acid D, which is the most notable toxic compound, especially when the fungus contaminates corn, and this poses a toxic risk due to its presence in the grain [177,178]. The presence of secalonic acid D in food can lead to safety issues because of its toxic and teratogenic effects. This highlights the need for strategies to prevent contamination in the food supply to safeguard human and animal health [177,179,180]. Despite its harmful effects, secalonic acid D has gained interest in drug development as it shows potential for killing cells in various types of cancer, including leukemia, lung cancer, and pancreatic cancer [181,182,183].

Oxalic acid is one of the main organic acids produced by *P. oxalicum*, which contributes to colonization and degradation of substrates. This organic acid is also recognized as a pathogenicity factor in various fungi as it can reduce pH, chelate calcium, and compromise plant cell walls, therefore facilitating infection [73]. Oxalic acid is involved in fungal pathogenesis and has been extensively studied in *Sclerotinia sclerotiorum* and *Botrytis cinerea*, where it serves as an essential component of pathogenicity [184,185,186]. This knowledge is applicable to plant-pathogenic *P. oxalicum.* Studies have shown that *P. oxalicum* produces oxalic acid in yam tuber tissues, suggesting that the organic acid is involved in the pathogen’s pathogenesis [187]. Although oxalic acid enhances virulence, it is not the sole organic acid contributing to pathogenesis [186].

## 7. *Penicillium citrinum*

*Penicillium citrinum* is distributed worldwide and has been found in a variety of sources including soil, indoor spaces, and food items. According to Houbraken et al. [188], *P*. *citrinum* is frequently found in tropical and subtropical soils but is present in smaller numbers in temperate soils. This species thrives under mesophilic conditions, with a minimum growth of 5 °C or slightly higher, a maximum just above 37 °C, and an optimal range of 26–30 °C. At 25 °C, the lowest water activity reported for *P. citrinum* growth is between 0.80 and 0.84, and it can grow in the pH range of 2–10 [2].

*Penicillium citrinum* is less recognized than *P. expansum*, *P. digitatum*, and *P*. *italicum* as a pathogen of postharvest rot. *Penicillium citrinum* causing postharvest rot has been recorded in several fruit crops, including citrus, pears, grapes, pomegranates, strawberries, tangelos, lychee, and star gooseberry, as well as in bulbs, such as onions and garlic, yams, and mushrooms. Additionally, *P. citrinum* has also been reported to cause leaf spot and ear rot in corn (Table 4).

*Penicillium citrinum* is recognized for its ability to produce citrinin and can generate substantial amounts of the mycotoxin in controlled laboratory conditions [207,208]. Citrinin is a common contaminant in a variety of crops and their derived products, often present together with other mycotoxins [209]. Due to the hepatotoxic and nephrotoxic properties of citrinin, the presence of *P. citrinum* in crops and during postharvest stages raises food safety concerns and poses potential risks to consumers [210,211].

While there is a limited number of studies directly associating citrinin production with the disease severity or virulence of *P. citrinum* on fruits and other crops, studies on *P. expansum* suggests that citrinin and other secondary metabolites might influence colonization, host responses, and competitive interactions in rotting tissues [100]. It is feasible that similar mechanisms may play a role in the virulence of *P. citrinum*.

In addition to citrinin, *P. citrinum* also synthesizes other secondary metabolites, including mevastatin, tanzawaic acids, quinocitrinines, and ergot alkaloids [212,213]. Tanzawaic acids have been shown to possess various biological activities, such as antimicrobial, anti-inflammatory, and antioxidant properties [212].

*Penicillium citrinum* is also recognized as a beneficial fungus. Certain strains of *P. citrinum*, particularly those identified as endophytes and those found in the rhizosphere, exhibit plant growth-promoting characteristics that facilitate nutrient absorption, enhance seedling development, and mitigate disease severity [214,215,216]. Furthermore, some strains of *P. citrinum* demonstrate antagonistic activity against plant pathogens and insects [217,218].

The dual roles of *P. citrinum* highlight the diversity among various strains. To effectively harness *P. citrinum* in agricultural applications, it is essential to thoroughly screen and assess beneficial strains, eliminating those that are pathogenic or toxigenic. Understanding the regulatory and genomic basis of both beneficial and harmful characteristics of *P. citrinum* is required for its safe use in sustainable agricultural practices.

**Figure 1:**
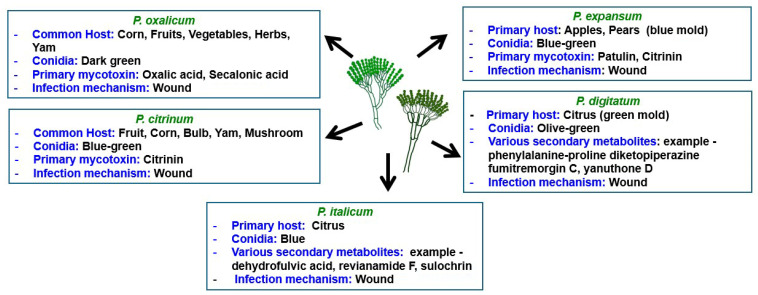
An overview of the host range, conidia colors, major mycotoxins, and infection mechanisms of *P. expansum*, *P. digitatum*, *P. italicum*, *P. citrinum*, and *P. oxalicum*.

Figure 1 provides an overview of the host range, conidia color, major mycotoxins, and infection mechanisms for *P. expansum*, *P. digitatum*, *P. italicum*, *P. citrinum*, and *P. oxalicum.*

## 8. Control Measures of *Penicillium* Rot

The management of *Penicillium* postharvest rot relies mainly on chemical control, and losses in quality during postharvest handling are often associated with this control measure. To effectively minimize postharvest rot, an integrated disease management strategy is essential. This strategy includes various approaches, including but not limited to the application of fungicides, sanitation or ensuring cleanliness in storage facilities, utilizing biological agents, and the use of resistant varieties when available [219]. Other approaches include the use of physical methods, essential oils, and plant extracts.

### 8.1. Fungicides

*Penicillium* rot can primarily be controlled by fungicides, including carbendazim, imazalil, thiabendazole, sodium orthophenyl phenate, fludioxonil, and pyrimethanil, which have been approved in various countries [220]. Some of these fungicides, such as thiabendazole, pyrimethanil, fludioxonil, and thiophanate methyl, are often used before storage to control postharvest pathogens [221]. Thiophanate methyl is regarded as the most effective fungicide for suppressing conidia germination and germ tube elongation in *P. expansum* [222].

Postharvest citrus fruit treatment is mainly controlled by pyrimethanil, imazalil, fludioxonil, and thiabendazole [223]. For pome fruits, thiabendazole has been widely used along with fludioxonil, pyrimethanil, and difenoconazole. According to Amiri and Ozturk [15], in postharvest applications for pome fruits and other fruit crops, fungicides for postharvest treatment should be applied promptly after harvest, as the risk of rot during storage increases with the time that passes following harvest. Imazalil, thiabendazole, pyrimethanil, and fludioxonil are fungicides commonly used in packinghouses before storage and are often used as a wash or drench to fruits [224,225].

The extensive application of fungicides has resulted in blue and green mold pathogens developing resistance. For example, the resistance developed by *P. expansum* populations has reduced the efficacy of certain fungicides [226]. As pathogens continue to build resistance and concerns about environmental and human health persist, rotating fungicides or incorporating various management strategies can help manage resistance [227]. Combining fungicides with other control methods such as cultural practices, proper sanitation, biological control, and reducing physical damage can improve the management of *Penicillium* rot and lessen the likelihood of developing fungicide-resistant pathogen populations [14].

### 8.2. Cultural and Sanitation Practices

Cultural and sanitation practices aimed at controlling *Penicillium* rot focus on minimizing the dissemination of conidia or spores and reducing the initial fungal population. In agricultural settings, particularly in orchards, the removal of rotten and mummified fruits helps reduce pathogen inoculum. Furthermore, implementing effective harvesting and handling techniques to minimize fruit bruising and mechanical damage is crucial in mitigating the risk of pathogen infections [15].

Postharvest sanitation practices have considerable effect on the control of *Penicillium* rot. It is important to clean and sanitize packing and storage room walls, floors and bins at least once in a season before using them to store new produce. Sanitation of the packing line from the dump tank to the sorters needs to be done regularly during the packing season. Sanitizers such as chlorine, chlorine dioxide, hydrogen peroxide, organic aids, and oxidizing water are available and have different efficacies and uses [15,228].

### 8.3. Biocontrol

Biocontrol offers an efficient and environmentally friendly alternative to fungicides, which are favored by consumers. To suppress growth and development of *Penicillium*, biocontrol agents applied include both yeasts and bacteria. Between 2016 and 2026, a search on Google Scholar for yeast and bacteria as biological control agents for blue and green molds yielded 17,700 results (as of 15 March 2026), highlighting that research on biocontrol of *Penicillium* rot pathogens is actively seeking effective biological solutions for managing postharvest diseases.

Yeasts are more frequently studied and used to control *Penicillium* postharvest infections than bacteria. This is due to the rapid growth of yeasts, their ability to quickly colonize fruit surfaces in unfavorable environments, their efficient use of host nutrients that suppress the growth of pathogens, and their enhanced resistance due to the production of an extracellular matrix, making them effective biocontrol agents [229,230]. The yeast *Candida oleophila* strain O is regarded as a highly effective and commercially available biological control agent for preventing postharvest rot in fruit such as apples and citrus [231,232]. This yeast has been reported to reduce rot diseases caused by *Penicillium* spp. by 62–98%, demonstrating efficacy comparable to the fungicides imazalil and thiabendazole [232]. Furthermore, Rovetto et al. [233] reported that *C. oleophila* significantly reduced the incidence of green mold symptoms in citrus throughout the postharvest supply chain, including harvesting, packinghouse processing, transportation, cold storage, and shelf-life stages. The study also revealed that the treatment stimulated citrus defense responses through the upregulation of several defense-related genes.

Several in vitro studies have shown the effectiveness of yeast as a biocontrol agent of *Penicillium* spp. associated with blue and green mold rot. Zhang et al. [234] demonstrated the effectiveness of the yeast *Wickerhamomyces anomalus* in controlling blue mold rot in pears by reducing spore germination and the germ tube length of the pathogen. In pears treated with 1 × 10^8^ cells mL^−1^, a disease incidence of 5.56% was recorded, in contrast to the 100% disease occurrence in the untreated control. In mandarin oranges, three yeast species, *W. anomalus, Metschnikowia pulcherrima*, and *Aureobasidium pullulans,* enhance the activity of two defense enzymes, peroxidase and superoxide dismutase, which help decrease the occurrence and severity of *P. italicum* [235]. A combination of three yeast species, *Meyerozyma caribbica, Metschnikowia zizyphicola*, and *Pichia rarassimilans,* effectively controlled blue mold pathogen and patulin degradation in apples [236]. In recent studies, Dudas et al. [237] and Zhang et al. [238] reported that *M. caribbica* effectively suppresses blue mold caused by *P. expansum* on several postharvest fruits by several mechanisms, including competition for nutrients and space and the production of antifungal volatile compounds such as phenylethanol. In addition, the yeast reduces patulin accumulation and enhances host resistance by stimulating defense-related enzymes and the accumulation of phenolic compounds, highlighting its potential as a biocontrol agent against postharvest blue mold. Similar to yeast, culture filtrates of filamentous fungi, *Trichoderma longibrachiatum*, *T. harzianum* and *Purpureocillium lilacinum,* could be effective as biocontrol agents against citrus blue and green mold pathogens [239,240].

In addition to yeasts, bacteria have shown potential as biocontrol agents against *Penicillium* spp., including *Bacillus subtilis* [241], *Pseudomonas fluorescens* [242,243], *Pantoea vagans* [244], *B. velezensis* strain S161 [245], *B. aryabhattai* AYG1023 [246], *Bacillus amyloliquefaciens* [247,248], various strains of lactic acid bacteria [249], and *Streptomyces* sp. [250]. Several in vitro studies highlight the potential of lactic acid bacteria (LAB) as antifungal agents and biopreservatives against postharvest *Penicillium* pathogens. For example, *Lactobacillus paraplantarum* CRL 1905 exhibited strong antifungal activity against *P. digitatum* and *P. italicum*, with its cell-free supernatant significantly inhibiting these major citrus pathogens. Similarly, the cell-free supernatant of *Lactobacillus plantarum* reduced *P. digitatum* growth on sweet oranges by up to 97.6% in vitro and 98.9% under storehouse conditions, demonstrating its potential as an alternative to synthetic fungicides [251]. In addition, LAB such as *Lactiplantibacillus plantarum* and *Lacticaseibacillus paracasei* have been shown to control the growth of *P. expansum* and remove patulin (up to 87.26%) in apple juice, supporting their role in food biopreservation [252]. These findings demonstrate the potential of LAB to effectively inhibit growth of green and blue mold pathogens and can be applied both in vitro and under postharvest storage conditions.

While biocontrol offers several benefits such as being non-toxic, environmentally friendly, generally safe and accepted by the public [253], this approach may face challenges in competing with conventional fungicides. Nonetheless, integrating biocontrol with fungicides and other control measures could be beneficial, particularly in situations where the pathogen population contains strains resistant to fungicides [254].

### 8.4. Integrated Control Measures

**Figure 2:**
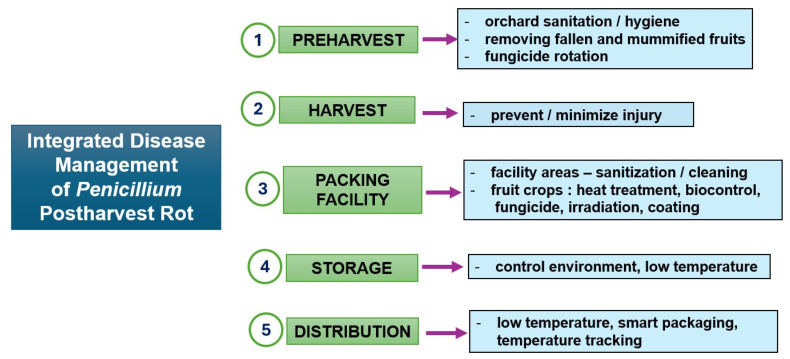
An overview of integrated disease management strategies for controlling postharvest *Penicillium* rot.

Utilizing a combination of control measures can result in a synergistic effect in managing blue and green molds, providing satisfactory control of postharvest pathogens in harvested crops [223]. By integrating control measures, the effectiveness and stability of individual treatments are enhanced, while also offering advantages that combine preventive and curative approaches. These integrated measures also help to reduce the time and cost associated with chemical treatments, as well as lower the dosage and phytotoxicity risks linked to other relevant treatments [255]. Figure 2 illustrates several approaches applied along the postharvest handling chain, which collectively contribute to reducing disease incidence and maintaining fruit quality

Fungicides are increasingly used at reduced rates in combination with physical methods, heat treatment, biological controls, and inorganic salt treatments to achieve effective control, while minimizing residues and resistance risk. Fungicides combined with hot-water treatment and food additives are effective in controlling postharvest rot. Hot-water treatment enhances fruit resistance to *P. digitatum* and *P. italicum* by inducing defense-related proteins, phytoalexins, and reactive oxygen species [256,257,258]. Integrated approaches, such as combining heat treatment with sodium bicarbonate, sodium benzoate, potassium sorbate, or carnauba wax, reduce rot of citrus and tangerines [259,260]. Notably, sodium benzoate combined with moderate heat treatment (50 °C for 60 s) achieved up to 90% reduction in green and blue mold rot incidence and acted synergistically with reduced fungicide doses [225].

Both UV-C and UV-B as non-ionizing irradiations have been extensively studied for controlling green and blue molds in citrus. UV-B is less damaging to citrus surfaces than UV-C [261] and can inactivate *P. digitatum* and *P. italicum* conidia at doses above 30 kJ m^−2^ [262]. X-ray and gamma irradiation are also effective in extending the shelf life of citrus fruits [263,264]. Additionally, LED blue light has shown the potential to reduce fungal growth by enhancing fruit resistance through secondary metabolite production and inhibiting fungal growth [265,266].

In Navel oranges, UV-C-irradiated orange essential oil and citral inhibited *P. digitatum* growth, although the level of inhibition was lower than that of the non-irradiated essential oil. Nonetheless, the treatment demonstrated a practical benefit, as it did not cause any visible injury or phytotoxicity to the fruit peel, highlighting its potential as a safe postharvest control alternative to fungicides [267]. The combined application of low-dose gamma irradiation (200–400 Gy) and *Pseudomonas fluorescens* effectively suppressed growth of *P. expansum* and reduced postharvest rot in apples. This combined treatment exhibited greater efficacy compared with individual methods, offering enhanced control of fungal growth while maintaining fruit quality during storage [268].

Combining chitosan coatings with essential oils is an effective method for controlling *P. italicum* and *P. digitatum* in fruits. This integrated approach forms a protective edible coating that exhibits enhanced antifungal properties, prolongs fruit freshness, and helps maintain fruit quality during storage. Essential oils such as spearmint, clove, and lemongrass are blended into the chitosan matrix, and their combined effect can surpass that of either ingredient used separately [269]. The application of chitosan with salicylic acid treatment reduced lesion size and the occurrence of rot disease while enhancing disease resistance against *P. digitatum*, resulting in an extended storage life with satisfactory postharvest quality in grapefruit [270]. Integrating glycol chitosan with the yeast *Candida saitoana* has been shown to be more effective in mitigating *P. digitatum* infections in lemons and oranges than using each treatment alone, achieving results comparable to the fungicide imazalil [271].

Integrating a crude extract of *Eugenia caryophyllata* with *Candida utilis* resulted in a reduction in both the incidence and severity of *P. digitatum* on citrus fruit. This combination of plant extract and biocontrol agent reduced the progression of green mold rot on citrus fruits without compromising fruit quality [272]. The application of garlic extract inhibited the growth and development of *P. digitatum* and *P. italicum*. Notably, the efficacy of garlic extracts was substantially enhanced when combined with vegetable oil, particularly with a 1% extract and oil treatment, which achieved complete (100%) control of both pathogens in Valencia oranges, comparable to the performance of the fungicides imazalil and quazatine [273].

The combination of plant extracts, *Saccharomyces cerevisiae*, and Generally Recognized as Safe (GRAS) salts has demonstrated potential as an alternative treatment for *P. digitatum* in citrus fruits [274]. Extracts from *Roylea cinerea*, *Murraya koenigii*, and *Mentha piperita* successfully inhibited the mycelial growth of the pathogen. Salicylic acid (0.25%), sodium bicarbonate (0.50%), and potassium sorbate (0.75%) completely inhibited the growth of *P. digitatum.* The application of *S. cerevisiae*, *R. cinerea* extract, and salicylic acid to citrus fruits reduced the incidence of green mold rot and disease severity, while also enhancing the nutritional quality of the fruit. These findings suggest that the integration of plant extracts, GRAS salts, and yeast constitutes an effective and environmentally sustainable strategy for controlling postharvest diseases in citrus fruits [274]. Table 5 shows a summary of several postharvest control strategies for *Penicillium* rot and their limitations.

Studies on the control methods for *Penicillium* blue and green mold rot are ongoing worldwide. A Google Scholar search conducted on 11 March 2026 found more than 17,100 publications (2016–2026) related to the management of *Penicillium* rot during storage, encompassing a wide range of approaches, including fungicides, biological control agents, essential oils, salicylic acid, chitosan, heat treatments, and other strategies. The substantial and continuously growing volume of studies over the past decade highlighted global research interest in these diseases, reflecting their significant economic impact and the ongoing challenges associated with their postharvest management in fruits and other crops worldwide.

Beyond the agricultural damage caused by these rot pathogens, certain species such as *P. expansum* present a public health concern due to their production of mycotoxins. Additionally, there is an increasing focus on developing eco-friendly and sustainable control methods, including the application of nanotechnology.

### 8.5. Nanotechnology

Nanotechnology employs nanoparticles such as chitosan, silver, and zinc oxide to manage fungal rot pathogens including blue and green molds. This is achieved through total removal of the pathogens, enhancement of antifungal properties, increased efficacy of biocontrol agents, and the development of protective coatings. These nanoparticles can be incorporated into edible coatings and essential oils to protect fruits or used in formulations with fungicides and beneficial microorganisms to enhance their antifungal properties and effectiveness [275,276]. Current studies are focused on assessing the efficacy of nanoparticles derived from various sources in mitigating the effects of infections caused by blue and green mold pathogens.

Cupric oxide nanoparticles (CuO-NPs) and silver nanoparticles (Ag-NPs) have demonstrated antifungal properties against *P. digitatum* and *P. italicum*, both of which are resistant to the fungicides imazalil, thiabendazole, and pyrimethanil [277]. Both types of nanoparticles disrupted membrane integrity and caused intracellular damage, while CuO-NPs further stimulated the generation of reactive oxygen species. Additionally, Ag-NPs diminished the residual infectivity of conidia even at lower concentrations and effectively controlled the green mold pathogens on artificially inoculated lemons, particularly fungicide-resistant strains. These results suggest that Ag-NPs are a promising nanomaterial alternative for antifungal treatment to safeguard fruits against postharvest citrus fungal pathogens [278].

Chitosan nanoparticles have been shown to enhance the resistance of apple fruit to blue mold rot caused by *P. expansum*, while also upregulating the expression of defense-related genes and preserving fruit quality. The optimal results were observed with chitosan nanoparticles at a concentration of 0.4 g/L for both artificially and naturally infected fruits. Fruit quality parameters, such as firmness, total soluble solids, and titratable acidity, were maintained in both infection conditions. The application of chitosan nanoparticles resulted in enhanced expression of genes linked to defense mechanisms [279]. Chitosan nanoparticles offer an environmentally sustainable and effective approach to control blue mold rot in apples and can be integrated into management strategies to maintain postharvest quality and extend the shelf life of fruits.

The application of essential oils for the management of postharvest rot is limited by their low solubility. Nevertheless, the encapsulation of essential oils within nanoparticles has emerged as a promising strategy to overcome these limitations. Chitosan nanoparticles loaded with lemon essential oil (CSNP-LO) have been successfully developed to combat infection by *P. expansum,* which causes blue mold rot of apple [280]. CSNP-LO inhibited conidia germination and reduced germ tube elongation. In postharvest apples, CSNP-LO reduced the incidence of blue mold rot and slowed lesion progression after a storage duration of 7 days. Additionally, the application of CSNP-LO enhanced the activity of defense-related and antioxidant enzymes in apples while preserving their quality. These findings demonstrated the potential of CSNP-LO as an innovative and practical method for controlling postharvest blue mold rot, hence improving the storage and shelf life of apples [280].

Studies by Maswanganye et al. [281], Dhanasekaran et al. [280] and Riolo et al. [282] highlight significant progress in postharvest delivery systems for essential oils, reflecting a shift from conventional aromatized coatings to more advanced nanoemulsion-based formulations. Maswanganye et al. [281] employed nanoemulsions formulated with oil from spearmint (*Mentha spicata*), illustrating the increasing application of nanotechnology to improve essential oil delivery. Dhanasekaran et al. [280] demonstrated that chitosan nanoparticles loaded with lemon essential could reduce apple decay by over 51%, significantly outperforming pure oil by triggering host resistance and improving controlled release. Riolo et al. [282] reported that a stabilized chitosan coating enriched with essential oils from cinnamon (*Cinnamomum verum*) and oregano (*Origanum vulgare*) effectively suppressed fungal growth through bioactive compounds such as (E)-cinnamaldehyde and carvacrol. This transition toward nanoemulsion systems enables a more uniform distribution of antimicrobial compounds and enhances the stability of volatile constituents, thereby improving the inhibition of green and blue molds caused by *P. digitatum* and *P. italicum*.

Through nanoemulsion delivery systems, the integration of essential oils into a chitosan matrix consistently preserves physicochemical features of treated fruits such as firmness, titratable acidity, and moisture content by creating a semi-permeable barrier that slows fruit respiration. Nanoemulsions of essential oils can enhance the sensory quality of the fruits by enabling the controlled release of volatile compounds, thereby preserving the fruit’s natural aroma without producing strong odors. Their small droplet size improves antimicrobial efficacy and supports better quality preservation during storage compared with conventional emulsions containing larger droplets [283]. In addition, the greater surface area and improved dispersibility of nanoemulsions contribute to enhanced antifungal activity and overall product quality maintenance [284].

Sensory analysis by Shah et al. [285] showed that pears treated with a chitosan–orange essential oil coating combined with ethylene scavengers achieved higher scores in appearance, texture, flavor, aroma, and overall acceptability than untreated fruits, indicating potential for extending shelf life while maintaining fruit quality. Maswanganye et al. [281] demonstrated that a chitosan (0.8%) coating loaded with 2% spearmint oil nanoemulsion provided complete inhibition of *P. digitatum* and *P. italicum* in soft citrus. By strengthening the coating’s structural matrix through high-shear homogenization (~250 nm droplets), this formulation offered an eco-friendly alternative with efficacy levels comparable to the fungicide Imazalil. These findings suggest that, when optimized for stability and sensory characteristics, chitosan–essential oil nanoemulsions can ensure high consumer acceptability of treated produce while offering a sustainable alternative to synthetic fungicides in the global postharvest supply chain.

Plant sources have also been used in the development of nanocomposites. A recent study examined a nanocomposite consisting of chitosan nanoparticles, artichoke extract, and AgNPs as an edible coating to inhibit growth of *P. italicum*, the causal agent of blue mold rot in oranges [286]. The results demonstrated that artichoke-mediated AgNPs, when combined with chitosan nanoparticles, form an antifungal nanocomposite suitable for use as an edible coating to protect citrus fruits against *P. italicum*. The interaction among the artichoke extract, AgNPs, and chitosan nanoparticles enhanced antifungal efficacy. Employing chitosan as a matrix may reduce the toxicity of nanoparticles and improve their stability or dispersibility for application in fruit coatings. This nanocomposite, comprising chitosan nanoparticles, artichoke extract, and AgNPs, may contribute to more sustainable management of postharvest blue mold rot in citrus, potentially reducing reliance on fungicides [286].

The study conducted by Tayel et al. [287] assessed the efficacy of edible nanocomposites composed of chitosan, fenugreek seed mucilage, and selenium nanoparticles (SeNPs) in protecting lemons from *P. digitatum* infection. Fenugreek seed mucilage facilitated the synthesis of SeNPs, which were then combined with chitosan to form nanocomposites, serving as antifungal edible coatings. The resultant composite demonstrated substantial antifungal activity with inhibition zones measuring 32.2 mm and an IC_50_ of 12.5 mg/mL, surpassing the effectiveness of the commercial fungicide imazalil. Application of this coating to lemons successfully inhibited *P. digitatum* infection over a period of 10 days. This study highlights the potential of chitosan, fenugreek seed mucilage, and SeNPs as sustainable control measures for postharvest protection and quality preservation in citrus fruits [287].

The use of nanoemulsions as a delivery system for biocontrol of blue and green mold pathogens represents a shift in postharvest technology. This is considered feasible because of their ability to improve stability, dispersibility, and controlled release of bioactive compounds, such as essential oils, which can enhance antimicrobial activity and extend shelf life [288]. By reducing droplet size, nanoemulsions improve the stability, dispersibility, and controlled release of antimicrobial compounds, including essential oils and microbial metabolites, enhancing antifungal efficacy while minimizing the required concentration of active agents [289]. This allows for more uniform coverage on fruit surfaces, better preservation of sensory and physicochemical quality, and extended shelf life compared to conventional formulations [290].

From a regulatory standpoint, the commercial application of nanoemulsion-based biocontrol agents must comply with existing food safety regulations. All formulation components should be approved as food-grade substances or recognized as environmentally safe. In addition, regulatory frameworks generally require comprehensive physicochemical characterization of nanoparticles, including parameters such as particle size distribution, stability, surface properties, and potential toxicity, to ensure their safety for consumers and the environment [284,291]. For example, regulatory authorities such as the European Food Safety Authority require detailed characterization of nanomaterials including particle size, solubility, and other physicochemical properties together with toxicological and exposure assessments before their approval for use in the food chain [292]. Furthermore, compliance with national and international regulations governing postharvest treatments is essential for facilitating market acceptance and ensuring the safe integration of nanoemulsion-based technologies within the global fruit supply chain.

Integrated disease management incorporating nanotechnology holds potential to enhance control of *Penicillium* postharvest rot. Nevertheless, the interaction among various control methods requires careful consideration to ensure synergistic efficacy and to prevent negative effects. Although integrated strategies for managing blue and green mold pathogens appear promising, their implementation in agricultural or commercial settings remains challenging. The main constraints include incompatibility among control methods, variable efficacy among different fruit types and other crops, and storage conditions that may impact the stability of the formulations [255]. Nanoparticle coatings also have limitations such as aggregation, high production costs, and safety concerns. The large-scale application is further complicated by the requirement for controlled environmental conditions and precise dosage. Regulatory uncertainty, lack of standardized testing methods, and limited consumer acceptance further hinder their utilization. Consequently, many nanotechnology-based control methods remain confined to laboratory or pilot stages and have not yet been widely adopted in the market [293,294].

## 9. Climate Change and Its Effect on *Penicillium* spp.

The impact of climate change on postharvest diseases caused by *Penicillium* spp. is expected to be substantial. These effects are largely driven by shifts in environmental factors that affect both the pathogen virulence and the host’s susceptibility [295]. The consequences of climate change, characterized by rising global temperatures, changes in precipitation patterns, and higher atmospheric CO_2_ levels, affect the pathogen and the host in complex ways, resulting in changes in the occurrence, severity, and management of postharvest diseases associated with *Penicillium* spp. [295].

### 9.1. Impact on Penicillium spp.

#### Geographic Expansion

Warmer temperatures enable *Penicillium* species, which were once restricted to particular region to extend their distribution into new, previously cooler areas. The geographic expansion of *Penicillium* is supported by various climatic factors; milder winters permit fungi to thrive in areas that were once too cold for survival, while increased moisture levels in the atmosphere allow for growth in regions that were too dry for fungal development [296].

Higher temperature and humidity can intensify the rates of fungal growth and colonization. Certain isolates might develop greater thermotolerance and virulence, enabling them to thrive in conditions that would restrict their growth. It is predicted that the temperature range for several *Penicillium* isolates may increase, with some thermotolerant and virulent strains demonstrating optimal growth at approximately 25–30 °C and having the ability to grow at 37 °C or even 40 °C [19,297]. In studies involving various *Penicillium* isolates from infected plants during both preharvest and postharvest periods, the growth temperatures were found to be around 25–30 °C, with numerous isolates demonstrating optimal growth at approximately 30 °C under normal ambient conditions [298]. The data suggest that increased thermal tolerance of *Penicillium* isolates could promote disease in warmer environments.

Elevated temperatures can enhance the virulence of pathogens by intensifying their life cycles and infection mechanisms. Increased CO_2_ levels can affect both the physiology of pathogens and the interactions between hosts and pathogens [299]. The higher thermotolerance observed in *Penicillium* isolates is often associated with increased virulence and an enhanced ability to infect and colonize host tissues in warmer conditions. Rising temperatures and increased CO_2_ concentrations, frequently interacting with humidity and water availability, may favor the growth of mycotoxigenic *Penicillium* species and the production of their mycotoxins, especially during times of water stress [300]. When temperatures exceed 30 °C in cooler areas, mycotoxins such as patulin and ochratoxin A may become more significant, as higher temperatures would facilitate the growth of *P. expansum* [301].

Climate change is expected to increase mycotoxin contamination in agriculture products as it provides favorable condition for fungal growth including mycotoxigenic *Penicillium*, driven by changes in temperature, humidity, and rainfall patterns. This may result in elevated mycotoxin levels in crops and the derived products, shifts in the geographical distribution of fungal species, and the emergence or increased prevalence of certain mycotoxins. The consequences of climate change include risks to food and feed safety, potentially leading to health-related complications and economic losses [301].

### 9.2. Fungicide Resistance

Shifts in weather patterns are becoming increasingly erratic due to climate change, increasing both the occurrence and severity of fungal diseases that affect crops during their growth and postharvest storage. To mitigate potential crop losses, it is expected that farmers are likely to rely on the use of fungicide to protect their yields, potentially leading to increased selective pressure for the development of resistant fungal strains [302]. Additionally, extensive application of azole fungicides in agriculture since the 1980s has contributed to the rise of azole-resistant fungal pathogens [303,304]. The occurrence of fungicide resistance is expected to escalate under climate-related stress, with *P. digitatum* showing decreased sensitivity to imazalil when temperature extremes coincide with fungicide application [106,305].

## 10. Future Perspectives and Conclusions

Although understanding of the causes of *Penicillium* rot and their management has improved considerably, future studies should focus on filling the gaps related to molecular, ecology, and translational aspects of *Penicillium*–host interactions. The main focus should be molecular aspects of pathogenicity mechanisms associated with necrotrophic infection. It is vital to further utilize comparative and functional genomics approaches to identify essential virulence determinants, such as cell wall-degrading enzymes, effectors, and regulatory pathways that facilitate colonization of the host plant [66,91]. The implementation of genome-editing technologies will enable direct functional validation of candidate genes and elucidate their roles in infection and aggressiveness. Integrating transcriptomic, proteomic, and metabolomic datasets across various stages of infection will be crucial for developing systems-level models of disease progression [22].

There is a need for more in-depth studies on the association between pathogenicity and secondary metabolites, particularly concerning patulin production in *P. expansum*. Future studies should focus on the regulatory mechanisms that connect mycotoxin production with signals from the host and conditions after harvest [19,21]. Gaining insight into how storage temperature, atmospheric conditions, and fruit physiology affect mycotoxin production will improve food safety risk and inform strategies to mitigate risks in commercial settings.

For future control measures of *Penicillium* rot in fruit crops, artificial intelligence (AI) is emerging as a transformative tool in postharvest management. Technologies such as machine learning, computer vision, robotics, and integration with the Internet of Things (IoT) are increasingly applied in sorting and grading, quality assessment, storage optimization, packaging, and cold chain logistics. AI-based systems have demonstrated potential in improving fruits classification, predicting shelf life, detecting early spoilage, and optimizing supply chain operations, thereby reducing human error and minimizing postharvest losses [306]. In parallel, the integration of IoT with distributed sensor networks and AI analytics enables the development of intelligent storage environments capable of actively preventing disease outbreaks. Smart storage systems equipped with wireless sensors continuously monitor critical parameters such as temperature, carbon dioxide, relative humidity, ethylene and volatile organic compounds, which serve as early indicators of pathogen activity, allowing timely adjustments or intervention to reduce the risk of *Penicillium* rot [307,308,309,310].

Studies concerning the host plant should focus on identifying the factors that contribute to fruit or crop resistance or tolerance against *Penicillium* infection. Even after harvest, fruits continue to exhibit active defense mechanisms, such as oxidative bursts and modifications to their cell walls [311,312], although the molecular basis and sustainability of these mechanisms remain inadequately understood. Employing induced resistance and defense mechanisms through various treatments such as physical, chemical, or biological offers a promising strategy for reducing the incidence of rot disease.

From an epidemiological perspective, population genetics, phylogeography and evolutionary studies are required for monitoring the emergence and dissemination of plant pathogens [313] which is applicable to studying plant-pathogenic *Penicillium*. Studies on population genetics and evolutionary dynamics of *Penicillium* will also give insights into virulence, fungicide resistance, and mycotoxin production. Furthermore, the integration of population genetic data with climate parameters and postharvest handling practices will facilitate predictive modeling of disease risk in the context of changing environmental conditions [314].

Future studies of *Penicillium* rot will also depend on translational research that connects basic findings with practical applications. The combination of rapid diagnostic methods [315,316], sensor-based detection technologies, and decision-support systems [317,318,319,320] will facilitate early detection and enhance disease management in the supply chains. In summary, interdisciplinary and global research initiatives are crucial for achieving sustainable control of *Penicillium*-related postharvest diseases and safeguarding both crop value and food safety.

In conclusion, *Penicillium* species continue to be among the most serious postharvest pathogens, not only on fruit crops but also other susceptible crops, leading to economic losses and food safety concerns mainly due to mycotoxin contamination. Climate change is likely to affect the epidemiology of *Penicillium* rot in the field and during postharvest. Understanding and anticipating environmental shifts is essential for developing adaptive storage practices and integrated management measures to protect crop quality and ensure food safety under changing climatic conditions.

## Figures and Tables

**Table 1:** Plant diseases associated with *Penicillium expansum*.

Crop	Disease	Country	References
Apple	Blue mold/Fruit rot	Uruguay	[28]
		Canada	[29]
		Apulia, Italy	[30]
		Pennsylvania, Oregon, USA	[31]
		Mount Lebanon, Lebanon	[32]
		Bishkek, Kyrgyzstan	[33]
	Fruit rot	South Africa (imported apples)	[34]
	Wet core rot	South Africa	[35]
		Shaanxi Province, China.	[36]
		Ceres, Grabouw, and Ermelo, South Africa	[37]
Pear	Blue mold	Korea	[38]
		South Africa	[39]
		Alcobaça, Portugal; Apulia, Italy	[40,30]
	Postharvest fruit rot	Bishkek, Kyrgyzstan	[41]
Citrus Kinnow mandarin (*Citrus nobilis* × *Citrus deliciosa*)	Fruit rot	Faisalabad, Pakistan	[42]
Lemon (*Citrus limon*)	Fruit rot	Beijing, China	[43]
Plum	Postharvest rot	South Africa	[44]
Nectarine	Postharvest fruit rot	South Africa	[44,45]
Grapes	Fruit rot/blue mold		
	Vineyard	Alentejo, Douro, Ribatejo and Vinhos Verdes, Portugal	[46]
	Storage	Copiapó and Nancagua, Chile	[47]
	Storage	Daejeon, Naju and Suwon, Korea	[48]
	Storage	Punjab province, Punjab	[49]
Grapes	Noble rot		
	During harvest and postharvest	Me’doc, Sauternes, Beaujolais, Loire Valley, Burgundy, France	[50]
	Drying room	Soave, Italy	[51]
Kiwifruit	Fruit rot	Prefecture of Kavala, Greece	[52]
		Jilin Province, China	[53]
		Lahore, Pakistan	[54]
		Prefecture of Kavala, Greece	[52]
Strawberry	Fruit rot	Not mentioned	[55]
Pomegranate	Postharvest fruit rot	Haryana, India	[56]
		Spain	[57]
		Alicante, Spain	[58]
Herb *Polygonatum odoratum* var. *pluriflorum*	Blue mold	Geumsan, Republic of Korea	[59]
Onion	Blue mold (storage)	Serbia	[60]
Ornamental bulb (iris, tulip)	Bulb rot	Washington State and Idaho	[61]
Perennial vine legume (*Apios mericana*)	Tuber rot	Muan, Jeollanam-do, Republic of Korea	[62]

**Table 2:** Plant diseases associated with *Penicillium digitatum*.

Crop	Disease	Country	References
*Citrus* spp.			
Citron (*Citrus medica*)	Fruit rot	Kunming, China	[113]
Mandarin orange (*Citrus reticulata*)		Central Valley, California	[114]
Nectarine	Postharvest fruit rot	South Africa	[45,44]
Plum	Postharvest fruit rot	South Africa	[44]
Ginger	Market and storage rot	Bahawalpur District, Pakistan	[112]

**Table 3:** Plant diseases associated with *Penicillium oxalicum*.

Plants/Crops	Disease	Country	References
Muskmelon (*Cucumis melo*)	blue mold	Songkhla Province, southern Thailand	[148]
		Minhang district, Shanghai, China	[149]
Grapes	noble rot (in drying room)	Soave, Italy	[51]
Mandarin orange (*Citrus reticulata*)	fruit rot	Wenzhou City of Zhejiang Province, China	[150]
Pineapple (*Ananas comosus*)	leaf spot	Leizhou Peninsula, China	[151]
Kiwi trees (*Actinidia chinensis* cv. Hongyang)	leaf spot	Xuzhou municipality, Jiangsu Province, China	[152]
Blue honeysuckle (*Lonicera caerulea*)	fruit rot	Harbin, China	[153]
Tomato	blue mold	Gyeongsangnam-do Agricultural Research and Extension Services, South Korea	[154]
	stem rot (blue mold)	Culiacan Valley, Mexico	[155]
	stem rot (blue mold)	Chiba Prefecture, Japan	[156]
	stem rot	Culiacan, Sinaloa, Mexico	[157]
Cucumber	stem and fruit rot	Lea Valley, Essex and Isle of Wight, England	[158]
	stem rot	Leamington area, southwestern Ontario	[159]
		Richmond, British Columbia, Canada	[160]
Corn	corn ears	Purdue University, USA	[161]
	corn ears	Bari, Italy	[162]
	seedling blight	Bat Dagan, Israel	[145]
	leaf blight	Southeastern Jiangsu, Nantong Municipality, China	[163]
Yam (*Dioscorea* spp.)	dry tuber rot	Southwestern Nigeria	[164]
Foshou Yam (*Dioscorea esculenta*)	tuber rot	Wuxue, Hubei Province, China	[165]
Yam (*D. rotundata*)	tuber rot	Idah Local Government, Kogi State, Nigeria	[166]
Herb (*Astragalus membranaceus*)	blue mold	Jilin Province, China	[167]
Herb (*Gastrodia elata*)	blue mold	Jilin Province, China	[168]

**Table 4:** Plant diseases associated with *Penicillium citrinum*.

Plants/Crops	Disease	Country	References
Oranges (*Citrus sinensis*)	Fruit rot	Brazil	[189]
Persian lime	Postharvest rot	San Pedro Lagunillas, Nayarit, Mexico	[190]
Akizuki’ pear	Postharvest rot	Hebei Province, China	[191]
Pomegranate	Postharvest rot	Southern Italy	[192]
Grapes	Blue mold (Vineyard)	Alentejo, Douro, Ribatejo and Vinhos Verdes, Portugal	[46]
	Blue mold (Storage)	Daejeon, Naju and Suwon, Republic of Korea	[48]
	Blue mold (Vineyard)	Nitra, Slovakia	[193]
Strawberry	Fruit rot	Qena city, Egypt	[194]
Chinese bayberry (*Myrica rubra*)	Postharvest rot	China	[195]
Tangelo (*Citrus* × *tangelo)*	Postharvest rot	China	[196]
Litchi	Postharvest rot	Limpopo Province and Western Cape Province, South Africa	[197]
Shengzhou plum fruits (*Prunus salicina* var. *taoxingli*)	Postharvest rot	Shengzhou, Zhejiang Province, China	[198]
Star gooseberry (*Phyllanthus acidus*)	Postharvest rot	Andhra Pradesh, India	[199]
Aonla/India gooseberry (*Phyllanthus officinalis*)	Postharvest rot	Sastri Market, Raipur	[200]
Onion	Storage rot	Sokoto, Nigeria	[201]
Garlic	Postharvest rot	Sahiwal and Lahore, Pakistan	[202]
Onion and Garlic	Postharvest rot	Nsukka, Enugu State, Nigeria	[203]
Yams (*Dioscorea rotundata,* *D. alata*)	Postharvest rot	Igwuruta town, Rivers State, Nigeria	[204]
Mushroom (*Dictyophora rubrovalvata)*	Green mold	Asuo village, Baiyun District Guiyang city, Guizhou Province, China	[205]
Corn	Ear rot	Peoria, Illinois, USA	[206]
	Leaf blight	Southeastern Jiangsu, Nantong Municipality, China	[163]

**Table 5:** Summary of control methods to manage *Penicillium* spp. causing blue and green mold rot.

Control Method	Description	Limitations
Fungicides (examples: imazalil, thiabendazole, pyrimethanil)	Commonly applied to postharvest fruit crops, these treatments effectively suppress fungal growth and conidia germination.	Reduced effectiveness due to resistance development, regulatory restrictions on fungicide use, and increasing concerns about chemical residues and consumer health.
Physical methods (examples: hot-water dips, irradiation, controlled storage conditions)	Directly reduces pathogen load while modifying the storage environment to slow fungal growth.	May induce heat or storage stress in fruits, shows limited effectiveness when applied alone, and may require specialized equipment.
Biocontrol agents: Yeasts (examples: *Candida oleophila*, *Metschnikowia* spp., *Pichia* spp.)	Compete with the pathogen for nutrients and ecological niches while also stimulating host defense responses.	Development of fungicide-resistant strains, increasing regulatory restrictions, concerns over chemical residues, and potential risks to consumer health.
Biocontrol agents: Bacteria (examples: *Bacillus* spp., *Lactobacillus* spp.)	Suppress pathogen development by producing antifungal metabolites and competing for nutrients and ecological niches.	Inconsistent efficacy among fruit commodities, challenges related to storage stability, and regulatory approval requirements.
Essential oils/Plant extracts (examples: mint, cinnamon, orange, lemon)	Antifungal compounds inhibit spore germination and mycelial growth.	Strong aroma or flavor may affect fruit quality, efficacy may vary, and high volatility can reduce persistence.
Chitosan-based coatings	Forms a protective physical barrier, induces host defense responses, and can be combined with essential oils or biocontrol agents.	May alter fruit appearance or texture, show reduced efficacy if improperly applied, and involve additional cost considerations.
Combination of methods (examples: biocontrol agent + coating; coating + essential oil)	Synergistic effects improve the overall efficacy of disease control.	May involve complex formulations, increased costs, potential regulatory challenges, and interactions that could reduce the effectiveness of individual components.

## Data Availability

No new data were created or analyzed in this study. Data sharing is not applicable to this article.
